# Marker-assisted backcross breeding for heat tolerance in bread wheat (*Triticum aestivum* L.)

**DOI:** 10.3389/fgene.2022.1056783

**Published:** 2022-12-08

**Authors:** Amasiddha Bellundagi, K. T. Ramya, Hari Krishna, Neelu Jain, P. Shashikumara, Pradeep Kumar Singh, Gyanendra Pratap Singh, Kumble Vinod Prabhu

**Affiliations:** ^1^ ICAR-Indian Institute of Millets Research, Hyderabad, India; ^2^ ICAR-Indian Agricultural Research Institute, New Delhi, India; ^3^ ICAR-Indian Institute of Oilseeds Research, Hyderabad, India; ^4^ ICAR-Indian Grassland and Fodder Research Institute, Jhansi, India; ^5^ ICAR-Indian Institute of Wheat and Barley Research, Karnal, India; ^6^ Protection of Plant Varieties and Farmers’ Rights Authority, Government of India, New Delhi, India

**Keywords:** heat tolerance, MABB, markers, backcross, early anthesis

## Abstract

Manipulation of flowering time for adaptation through natural or genetic approaches may combat heat-stress damage that occurs at the reproductive stages in production conditions. HD2733, a popular wheat variety of the eastern plains of India, is largely sensitive to heat stress. Therefore, the current study aims to improve heat tolerance of HD2733 by introgression of QTLs associated with early anthesis and high kernel weight linked to markers X*barc186* and X*gwm190*, respectively, through marker-assisted backcross breeding (MABB) from a tolerant donor, WH730. A total of 124 simple sequence repeat (SSR) markers distributed evenly across the genome were used for the background selection. The alleles of *Xbarc186* and X*gwm190* were fixed in BC_2_F_1_ and BC_1_F_2_ generations by selecting individual plants heterozygous for both marker loci and backcrossed with HD2733 and simultaneously selfed to generate BC_2_F_1_ and BC_1_F_2_ populations, respectively. Furthermore, the selected BC_1_F_2_ were selfed to generate the BC_1_F4 population. By background screening, a total of 39 BC_2_F_3_ and 21 BC_1_F_4_ families homozygous for the targeted QTLs with 90.9–97.9% and 86.8–88.3% RPG recoveries were selected. The best performing 17 BC_2_F_3_ and 10 BC_1_F_4_ lines were evaluated for various morpho-physiological traits. Phenotypic evaluation and multi-location trials of the introgressed lines under late sown conditions led to the selection of three promising lines with early anthesis and higher grain yield. The improved lines will serve as an excellent genetic material for functional genomics and expression studies to understand the molecular mechanisms and pathways underlying the stress tolerance.

## Introduction

Wheat is an important cereal crop in terms of global annual acres grown and tonnage harvested. According to FAO statistics, 772.64 million tons of wheat is harvested from 220.4 mha area in the world (FAO, 2020). High temperature stress during the anthesis period can not only reduce grain yield but also the quality of wheat (Stone and Nicolas, 1994; Langridge and Reynolds. 2021). Climate change is likely to increase the problem of high temperature stress to wheat production in many parts of Asia (Ortiz et al., 2008; Sun et al., 2021). The effects of climatic change are highly noticed in major wheat-growing regions of India, with frequent heat waves and earlier onset of higher temperatures. Furthermore, a substantial area under wheat cultivation is subjected to heat stress due to delayed planting (Joshi et al., 2007).

The central zone and north-eastern plain zone of India encompasses nearly 7 mha of wheat-growing area which is prone to high-temperature stress and nearly 13.5 mha of wheat-growing area is affected by heat stress (Bhusal et al., 2017). Increased ambient temperature above 30°C during the grain-filling period is a major threat for wheat productivity and grain-quality standards (Wardlaw and Moncur, 1995; Rane and Nagarajan, 2004) by affecting the duration and rate of grain development (Dias and Lidon, 2009; Pandey et al., 2013). The North Eastern Plain Zone (NEPZ) covers more than 33% of the wheat-growing area with a predicted yield potential of 4.5–5.0 t/ha. However, farmers in this zone realize only 2.5 to 3.0 t/ha of production, due to late sowing in the end of November or the first week of December, which leads to the exposure of the crop to high temperature during the reproductive growth period (anthesis to grain maturity) causing reduced spikelet fertility and grain filling, thereby reducing the yield (Shashikumara et al., 2022b). Manipulating the flowering time either naturally or through genetic approaches may combat heat stress damage during the reproductive stages (Jagadish et al., 2020). Thus, the development of wheat cultivars with built-in heat-tolerant traits such as early anthesis and high kernel weight are rewarding in boosting wheat production under high-temperature regimes. Earliness in wheat acts as an adaptive mechanism to avoid heat stress and has been observed in the release of early maturing varieties and heat-tolerant wheat in South-Asia (Mondal et al., 2016). Improving heat tolerance in plants through traditional methods of breeding is comparatively difficult as heat tolerance is a complex trait manifested by various yield and physiological adaptive traits (Manjunath et al., 2021). Selection based on phenotyping alone is tricky and time-consuming in case of complex traits affected by the environment on its expression. Heat tolerance is a quantitative trait which is influenced by prevailing environments. Hence, selection for such traits using phenotyping tools will be tricky and difficult in segregating generations. Identification of genomic regions governing such adaptive traits could be helpful in improving yield stability under stress using molecular marker-assisted transfer of genes/QTLs to improve thermo-tolerance.

Although the application of conventional plant breeding programs has a significant impact in improving the productivity under marginal wheat-growing environments (Manu et al., 2020), genetic improvement needs a more systematic use of physiological and molecular genetic approaches. Molecular markers are highly efficient in QTL identification and introgression of QTLs into the required genetic background through marker-assisted backcrossing (Puttamadanayaka et al., 2020). Marker-assisted backcross breeding is one of the best breeding methodologies to accelerate the improvement of varieties by adopting marker-based selection of genes/QTLs governing desirable traits (Hospital, 2003; Shashikumara et al., 2022a). Marker-assisted backcross breeding has successfully been demonstrated in various crops such as rice (*Oryza sativa*) (Neerja et al., 2007; Singh et al., 2013), wheat (*Triticum aestivum*) (Somers et al., 2004; Zhou et al., 2005), maize (*Zea mays* L.) (Tamilkumar et al., 2014; Muthusamy et al., 2015), and so on for biotic and abiotic stresses. In wheat, MABB was efficiently used for high molecular weight glutenins (de Bustos et al., 2001), grain protein content enhancement (Davies et al., 2006), drought tolerance (Rai et al., 2018), and pre-harvest sprouting tolerance (Torada et al., 2008).

Most of the heat stress adaptive traits are polygenic in nature. QTLs identified for physiological and yield traits were also found to contribute to improving adaptation under heat stress (Pinto et al., 2010; Kadam et al., 2012; Kumar et al., 2012; Ramya et al., 2021). Despite the availability of a large number of QTLs for heat stress-governing traits, few QTLs have been validated and fewer have been used in practical wheat-breeding programs. Hence, the present study was undertaken to transfer available heat-tolerant QTLs from donor parent WH730, an identified heat stress-tolerant line into a well-adapted, high-yielding variety of HD2733. HD2733 is one of the popular varieties, cultivated in more than 30% of area in the NEPZ and having a high indent for breeder seed requirement (www.iiwbr.org.in), but it is heat stress susceptible as there is significant reduction in the yield potential under high-temperature stress.

To improve HD2733 for heat tolerance and to overcome the yield reduction, two QTLs were targeted for transfer through MABB which is known as the most eco-friendly and sustainable approach to develop stress-tolerant varieties. The first QTL was transferred for early anthesis linked with marker X*barc186* (Pinto et al., 2010) and the second major QTL linked with marker X*gwm190* was targeted for kernel weight and grain yield under heat stress (Mohammadi et al., 2008). The improved NILs possessing targeted QTLs were further analyzed for the performance of QTLs in homozygous generations.

## Materials and methods

### Plant material and experimental site

The recurrent parent HD2733, a high-yielding variety, was released for the North Eastern Plains Zone (NEPZ) of India under irrigated timely sown conditions. It is double dwarf (82 cm), resistant to leaf rust and leaf blight, medium to early maturing (130–135 days) with average yield of 5.0 t/ha under timely sown and irrigated conditions. The seeds of HD2733 were obtained from wheat-breeding section, IARI, New Delhi, India. WH730 (IC546937) (derived from a cross of CPAN2092/Improved Lok1), developed by Chaudhary Charan Singh Haryana Agricultural University, Hissar, Haryana, India, was used as the donor parent. The variety had a higher grain yield, low heat susceptibility index, high kernel weight, membrane thermo-tolerance, and grain number under high-temperature stress (Dhanda and Munjal, 2012; Gupta et al., 2013). The experiment was conducted at the Division of Genetics, Indian Agricultural Research Institute, New Delhi, India. All the package of practices recommended for bread wheat crop was followed.

### Molecular marker analysis

Leaf samples were collected from 25- to 30-day-old seedlings for DNA isolation using a protocol as described by Prabhu et al. (1998). PCR was performed in a 10-µl reaction mixture containing 10–25 ng of template DNA, 1 µl 10X buffer (containing 500 mM KCL, 15 mM MgCl_2_, 200 mM Tris HCl, pH 8.3), 0.4 µl of 10 mM dNTPs, 1 µl each of 5 mM forward and reverse primers, 0.4 µl of *Taq* DNA Polymerase (2 U/µl), and double-distilled water to make up the volume to 10 μl, using a 96-well thermal cycler. The PCR program was as follows: initial denaturation for 5 min at 94°C, each cycle comprised 1 min, denaturation at 94°C, 1 min annealing at 55-60°C (depending upon the X*gwm*/X*wmc* primer), and 1 min extension at 72°C with a final extension for 10 min at 72°C at the end of 45 cycles. For X*barc* and X*cfd* series, the thermo-cycling program included initial denaturation for 5 min at 94°C, followed by 30 cycles where each cycle comprised 30 s of denaturation at 94°C, 30 s of annealing at 60°C, and 30 s of extension at 72°C with a final extension for 10 min at 72°C. The PCR products were analyzed by electrophoresis on 3.2% agarose/metaphor^TM^ gel stained with ethidium bromide and were documented using Alpha Imager 1220 (Alpha Innotech, CA, United States).

### Marker-aided development of improved lines

**FIGURE 1:**
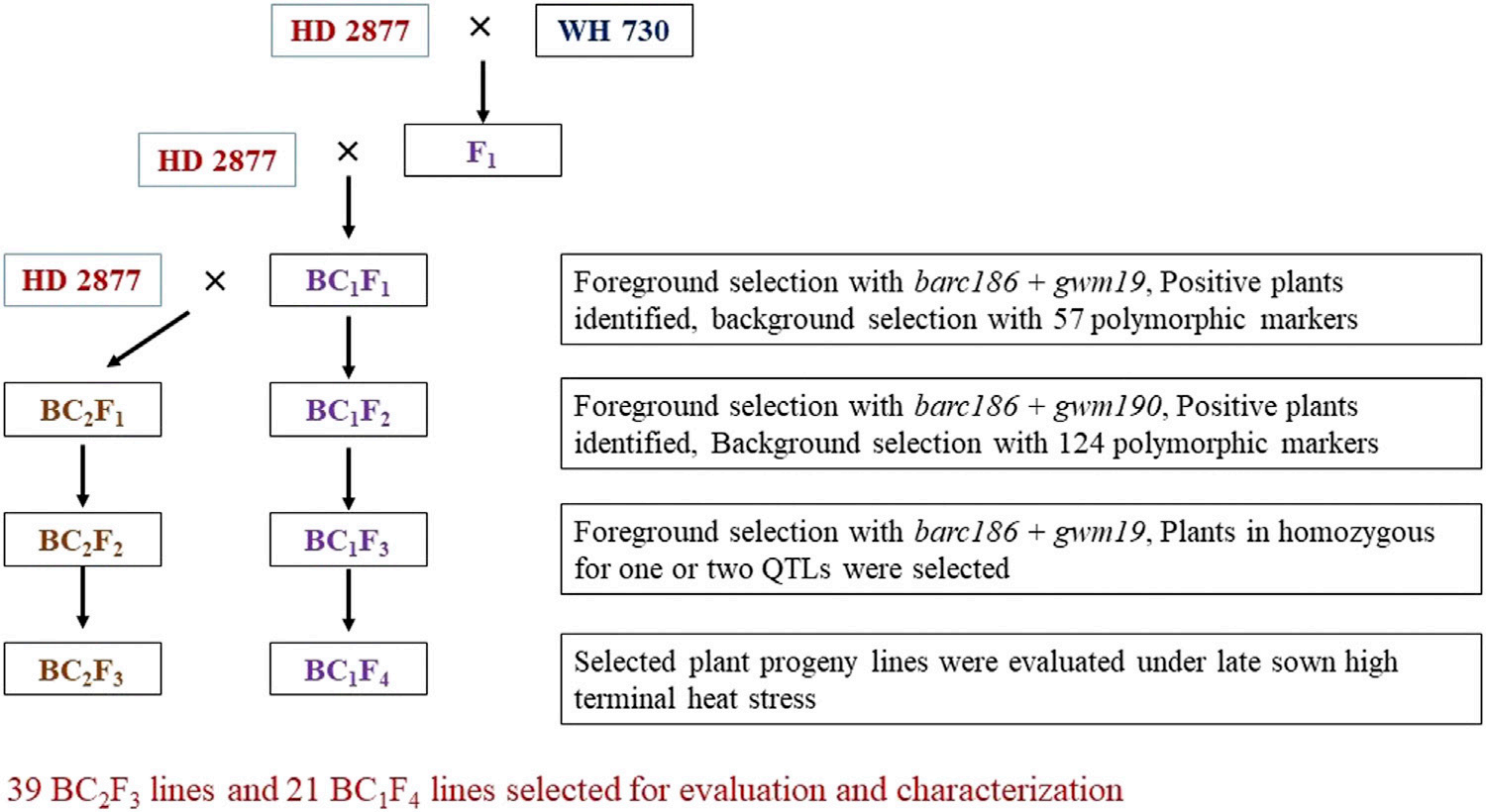
MABB scheme for the improvement of HD2733 using WH730 as a donor for heat-tolerance QTLS.

During the rabi season, crosses were affected by hand emasculation of HD2733 and pollination with WH730 pollens to generate sufficient F_1_ seeds. F_1_ plants with confirmed hybridity through foreground markers were backcrossed to HD2733 to produce BC_1_F_1_s and subsequent generations were forwarded as per the MABB scheme presented in Figure 1. The scheme includes a three-step selection strategy in each backcross generation: (1) foreground selection for the target QTLs using linked SSR markers; (2) a two-phase background selection using 124 SSR polymorphic markers, 57 of these markers (nearly half the set of the total polymorphic markers) were used for background scoring in BC_1_F_1_, and the remaining 67 polymorphic markers and the markers heterozygous in BC_1_F_1_ were used in BC_2_F_1_ to select plants homozygous for recurrent parent alleles at the maximum number of loci to increase the recurrent parent genome (RPG) recovery and genome coverage; and (3) stringent phenotypic selection for agro-morphological traits and physiological traits to accelerate the recurrent parent phenome (RPP) recovery.

The desirable BC_1_F_1_ plants obtained as per the three-step selection strategy explained previously were backcrossed with HD2733 to develop BC_2_F_1_ lines and advanced with two generations of selfing to obtain BC_2_F_2_ (Off-season nursery) and BC_2_F_3_ lines. Simultaneously, BC_1_F_1_ plants were selfed to generate BC_1_F_2_ generation and advanced to obtain BC_1_F_3_ (off-season nursery) and BC_1_F_4_ generations. Foreground and background selections coupled with phenotypic selection were again carried out in the BC_2_F_1_ and BC_1_F_2_ generations; the SSR markers which were homozygous and fixed were not considered for background selection. QTL-positive plants with high RPG and RPP were advanced to BC_2_F_2_and BC_1_F_3_ generations. The donor QTLs were confirmed in BC_2_F_2_ and BC_1_F_3_ and these were subsequently selfed to generate BC_2_F_3_ and BC_1_F_4_ families.

### Foreground selection

Foreground selection for the targeted QTLs was carried out using QTL-linked markers, X*barc186* (Pinto et al., 2010) and X*gwm190* (Mohammadi et al., 2008), respectively (Table 1). The X*barc186* marker reported in a reciprocal cross of Seri M82/Babax-derived RILs population governs early anthesis, causing early maturity with 6.4% phenotypic variance, and the X*gwm190* marker reported in the RIL population of a cross between MTA16/Kauz governs high kernel weight and grain yield under stress with 44.3% phenotypic variance. QTL mapping for heat stress-related traits had also been carried out in the WH730 x HD2733 mapping population in our laboratory at IARI. In this earlier study, QTL linked to early heading and anthesis (X*barc186*) was mapped on chromosome 5A and QTL for grain yield (X*gwm190*) mapped on chromosome 5D (Sun et al., 2021). These SSR markers were also validated in the segregating BC_1_F_2_ population (296 plants) using single-marker analysis during the present study.

**TABLE 1:** Details of the markers used in the foreground selection of backcross-derived lines.

QTL	Marker	Forward primer	Reverse primer	Chr	Reference	R^2^ value	*p*-value
Days to anthesis	Xbarc186	5′ GGA​GTG​TCG​AGA​TGA​TGT​GGA​AAC 3′	5′ CGC​AGA​CGT​CAG​CAG​CTC​GAG​AGG 3′	5A	[25]	0.089	0.001
Grain yield under stress	Xgwm190	5′ GTG​CTT​GCT​GAG​CTA​TGA​GTC 3′	5′ GTG​CCA​CGT​GGT​ACC​TTT​G 3′	5D	[28]	0.245	1.13E-08

BC_1_F_1_ plants heterozygous for the X*barc186* and X*gwm190* markers at both loci were selected for making backcrosses with HD2733 to generate the BC_2_F_1_ population. Simultaneously, the selected BC_1_F_1_ plants were selfed to generate the BC_1_F_2_ population and advanced up to BC_1_F_4_ generation. A similar strategy was used to select individual plants in the BC_2_F_1_ generation and selfed to get BC_2_F_2_ and BC_2_F_3_ generations (Figure 1).

### Background selection

A parental polymorphic survey was carried out between donor and recurrent parent by screening parents for 1,350 microsatellite markers (Pestsova et al., 2000; Gupta et al., 2002; Somers et al., 2004; Kadam et al., 2012; Röder et al., 1998). Initially, in the BC_1_F_1_ generation, 57 polymorphic markers were used to screen the selected individual plants for recipient parental genome recovery and donor parent allele replacement at other regions of the chromosomes except at the targeted regions; later on, in BC_2_F_1_, BC_2_F_2_, BC_2_F_3_, BC_1_F_2_, and BC_1_F_3_ generations, 67 additional SSRs were used for background selection to calculate genome recovery. A total of 124 molecular markers differentiated the parents at the genome level, which were used for background selection (Supplementary Table S1). The genome contribution of the parents in the improved lines was analyzed and depicted using the software Graphical Genotypes (GGT) Version 2.0 (van Berloo, 1999). The recurrent parent genome recovery (RPG) percentage was calculated by using the formula RPG (%) = (R + 1/2H) × 100/P, where R is the total number of markers homozygous for a recurrent parent allele, H is the total number of markers which remained heterozygous, and P is the total number of polymorphic markers used in the background selection program. Chi-square (χ2) test of goodness of fit with one degree of freedom was used to test the observed and the expected segregation ratio of the targeted QTLs.

### Evaluation of derived lines for targeted trait improvement and other morpho-physiological traits

The experiment was laid out in an augmented design with four replications of parental checks in BC_2_F_3_ lines and three replications in BC_1_F_4_ lines. Two rows of each genotype were planted in a plot size of 0.46 × 2.5 m keeping 23 cm between rows. The standard cultivation field practices followed in wheat under normal (mid of November) and late sowing (second quarter of December) conditions to expose them to heat stress were followed precisely. The IARI research farm had calcic xe-rosol type of soil with a mean maximum temperature of 26.6°C and a mean rainfall of 2.9 mm during the wheat-growing seasons. Parental lines were raised under normal sowing and also at late sowing conditions for an accurate comparison of the derived lines with parents. Data for targeted traits and different morpho-physiological traits, namely, days to flag leaf emergence (FLE), days to heading (DH), days to anthesis (DA), and days to maturity (DM), were recorded on a visible basis, number of productive tillers per plant (tillers/pl), number of spikelets per spike (spk/sp), 1000-kernel weight (TKW), number of grains per 5 spike, grain yield per 5 plants, biomass, and harvest index (HI) were measured on five plants. Observations on days to flag leaf emergence, days to heading, and days to maturity were measured by counting the days from date of sowing to the respective stages of the crop. Traits such as plant height, spike length, peduncle length, number of productive tillers per plant, number of spikelets per spike, and number of grains per 5 spike observations were taken on 5 randomly selected plants and their means were used for analysis. Biomass was recorded as above ground weight of the five selected plants.

Among the physiological traits, the normalized difference vegetation index (NDVI) was measured with a field-portable Greenseeker at three growth stages (late boot stage, early milky stage, and late milky stage). The chlorophyll content was measured using a Minolta SPAD-502 chlorophyll meter at the three stages of growth. Stomatal conductance was measured using Decagon: SC-1 hand-held porometer at two growth stages (late-boot stage and early milky stage). Early ground cover was measured following the method described by Mullan and Reynolds (2010). With the use of a compact digital camera, images were acquired without using the zoom function at 25 days after germination, one image per plot was taken from a distance of constant 1 m height, and the digital photographs were processed. A hand-held infrared thermometer (Kane May Model Infratrace 8000, United States) was used to estimate the canopy temperature. Two measurements per plot nearly 0.5 m from the edge of the plot and approximately 1 m above the canopy were recorded. Membrane stability index (MSI) was estimated according to the method of Sairam et al. (1997). Leaf material (100 mg) was taken in test tubes having 10 ml of double-distilled water. Initial (C1) (40°C) and final (C2) (100°C) conductivities of the solution were noted on a conductivity bridge (Century, Water soil analysis kit, CMK 751). MSI was calculated as follows: MSI = [1 − (C1/C2)] × 100. Traits such as stomatal conductance and canopy temperature were measured on clear sunshine days at 11 a.m. to 12 p.m. h. CT and NDVI were measured two times a day, 11.00–11.30 a.m. and 1.00–1.30 PM. All physiological characters were measured at three developmental stages: late-boot stage, early milk stage, and late milk stage, which were considered as important and sensitive stages to heat stress.

The improvement of backcross-derived lines for the targeted traits and contribution of other morpho-physiological characters to yield under high-temperature stress was tested for significance (t-test at *p* < 0.05) by using critical difference at 5 per cent level of significance (CD5%). The Anderson Darling test was studied to know the distribution pattern of lines in each population. Correlation coefficients were studied to determine the effect of other traits on days to anthesis and grain yield. Analysis of variance (ANOVA) for the augmented design was studied in BC_2_F_3_ and BC_1_F_4_ progenies. The number of selected genotypes was further reduced in subsequent generations on the basis of improved agronomic performance over the recurrent parent. The 27 selected BC_2_F_4_ (17) and BC_1_F_5_ (10) families were planted next season in an alpha-lattice design that consisted of 4 blocks with 7 plots/blocks. The two replications were planted in three rows with a gross plot size of 0.63 × 2.5 m, with rows at 23 cm apart under late sown conditions (second quarter of December), and data for DA and yield traits were recorded. From these 27 lines, the selected 8 homozygous lines were evaluated at three locations, namely, Delhi, Pusa Bihar, and Pune under a net plot size of 7.2 sq. m each. Pusa Bihar (north-east India) and Pune (central India) represent the target locations for a heat-stress environment.

## Results

### Development of NILs using foreground and background selections

#### Genotyping and selection in BC_1_ generation

A total of 760 individual BC_1_F_1_ plants were tested for the presence of foreground markers and 266 plants were selected based on both phenotypic similarity to the recipient parent HD2733 and presence of foreground markers, X*barc186* and X*gwm190*. Out of the 266 selected plants, 40 plants were positive for the X*barc186* marker and 39 plants for the X*gwm190* marker, along with 187 plants positive for both the markers. Background screening using 57 polymorphic makers revealed a recovery percentage range from 67.3% to 75.4%. A total of seven plants showing heterozygous nature for both foreground marker loci, high RPG recovery of 74.5%–75.4%, and higher phenotypic similarity with a recurrent parent were used to make backcrosses to generate BC_2_F_1_ and selfed to produce BC_1_F_2_ generation. The details of the plant population in each generation, RPG recovery, and number of plants selected are given in Table 2.

**TABLE 2:** Number of plants selected and the recurrent parent genome recovery obtained in each of the backcross generations.

Generation	Total no. of plants obtained	Plants selected based on phenotypic similarity to RP	Markers used for background selection	% RPG in the QTL positive and high phenotypic similarity to RP plants	Plants selected after foreground, background, and phenotypic selection	% RPG in selected plants
BC_1_F_1_	760	—	—		7	
QTL-positive plants with 1 X*barc186*	377	40	57	67.3%–75.4%		74.5%–75.4%
2. X*gwm190*	374	39	57			
3. Both markers	187	187	57			
BC_2_F_1_	356	—	—			
1. X*barc186*	169	37	124	83.33%–94.44%	10	88.60%–94.44%
2. X*gwm190*	167	30	124		8	
3. Both markers	81	52	124		8	
BC_2_F_2_	800	Phenotypic selection not practiced at off-season nursery at Dalang maidan, Lahaul-Spiti, HP, India			39	
1. X*barc186*	383		124	89.73%–96.87%	12	90.83%–96.87%
2. X*gwm190*	417		124		21	
3. Both markers	186		124		6	
BC_2_F_3_	39	39	—		17	
1. X*barc186*	12		124	90.90%–97.90%	6	93.90%–97.90%
2. X*gwm190*	21		124		9	
3. Both markers	6		124		2	
BC_2_F_4_	17	17	—	—	6	93.90%–97.90%
BC_1_F_2_	296	68			21	
1. X*barc186*	70	27	124	86.84%–88.35%	7	86.84%–88.35%
2. X*gwm190*	68	24	124		10	
3. Both markers	20	17	124		4	
BC_1_F_3_	21	—			21	
1. X*barc186*	7		—	86.84%–88.35%	7	86.84%–88.35%
2. X*gwm190*	10				10	
3. Both markers	4				4	
BC_1_F_4_	21	10			10	
1. X*barc186*	7		124	92.0%–92.80%	4	92.0%–92.80%
2. X*gwm190*	10		124		3	
3. Both markers	4		124		3	
BC_1_F_5_	10	10	—	—	2	92.0%–92.80%

To examine the phenotypic expression of the two targeted QTLs, segregating the BC_1_F_2_ population (296 plants) was done for validation with linked SSRs through single-marker analysis. The results showed QTL for days to anthesis, located on chromosome 5A (co-segregated with the X*barc186* marker) showing a phenotypic variance of 8.9% (R^2^ = 0.089), and QTL for grain yield under stress, located on chromosome 5D, (co-segregated with the X*gwm190* marker) showed 24.5% (R^2^ = 0.245) phenotypic variance under high-temperature stress (Table 1).

### Genotyping and selection in BC_2_ generation

A total of 356 BC_2_F_1_ plants derived from seven plants selected in BC_1_F_1_ were screened with X*barc186* and X*gwm190* markers linked with QTLs of interest. A total of 119 plants, which includes 52 plants with both QTLs, 37 plants with one QTL linked to X*barc186* and 30 plants, and with another QTL linked to X*gwm190*, were screened for background recovery. The SSR marker loci which were heterozygous in BC_1_F_1_ were used for screening again to know the replacement of the donor parent allele by the recipient parent allele at the respective locus. There were 67 additional markers used for background screening of BC_2_F_1_ plants along with 57 markers already used in BC_1_F_1_. Based on the results of background screening, lines having a comparatively high recurrent parent genome recovery and phenotypic similarity of the plants with recurrent parent also targeted the trait similarity with donor parent allele; a total of 26 BC_2_F_1_ plants with RPG ranging from 88.60% to 94.44% were selected for the advancement to BC_2_F_2_ generation.

The segregating BC_2_F_2_ generation (800 plants) of the selected 26 BC_2_F_1_ plants were screened for the presence of targeted trait QTLs using linked markers. 59 plants homozygous for donor parent allele were used for screening with background markers which were heterozygous in BC_2_F_1_-selected individual plants. Finally, 39 plants with a higher RPG per cent ranging from 89.73% to 96.87% were selected. Out of 39 plants, six plants were with both QTLs, 12 plants were with X*barc186*-linked QTL, and 21 plants with X*gwm190* marker-linked QTL. The selected homozygous BC_2_F_3_ families of the 39 selected plants were subjected to foreground selection for the confirmation of the targeted QTLs. An improvement in the RPG recovery per cent from 90.90% to 97.90% (39 BC_2_F_3_ lines) was observed in the background selection. After evaluation of 39 plants for targeted traits and other morpho-physiological traits, 17 lines were finalized for advancement.

### Genotyping and selection in selfed BC_1_ generations

A total of 68 BC_1_F_2_ plants containing single or both QTLs in the homozygous condition were selected for background screening. Based on high RPG (ranging from 86.84% to 88.35%), high RPP, and targeted trait similarity with donor parent allele, a total of 21 BC_1_F_2_ plants (Table 2) were selected for advancement. The selected plants were selfed to generate BC_1_F_3_ and BC_1_F_4_ homozygous families.

### Chi square test for Mendelian segregation of QTLs

The chi-square test is done to test an expected ratio of 1:1 segregation for each QTL separately and also for the combination of QTLs in the BC_1_F_1_ and BC_2_F_1_ generations. It was established that the observed frequency of QTL-positive and -negative plants was in accordance to the Mendelian segregation pattern with an expected ratio of 1:1 and 1:1:1:1 for single and two QTLs, respectively (Table 3). In BC_1_F_1_ generation, the calculated χ^2^ values for *qAnth* (0.0473), *qGY (s)* (0.1924), and for combination of both QTLs (0.2834) (at *p* = 0.05 level of significance) were non-significant, agreeing with the null hypothesis of no difference. In BC_2_F_1_ generation, the calculated χ^2^ values (0.9111 for *qAnth*, 1.3594 for *qGY(s)*, and 1.1114 for a combination of both QTLs) were again non-significant (Table 3).

**TABLE 3:** Chi-square (χ^2^) test for QTL segregation in backcross generations.

Generation	QTL	QTL +ve plants		QTL −ve plants		Total no. plants	Observed ratio	Expected ratio	Total χ^2^ value at *p* = 0.05
BC_1_F_1_	*qANTH*	377		383		760	1:1	1:1	0.0473
	*qGY(s)*	374		386		760	1:1	1:1	0.1924
	*qANTH+* qGY(s)	187	187	190	196	760	1:1:1:1	1:1:1:1	0.2834
BC_2_F_1_	*qANTH*	169		187		356	1:1	1:1	0.9111
	*qGY(s)*	167		189		356	1:1	1:1	1.3594
	*qANTH+ qGY(s)*	85	85	88	97	356	1:1:1:1	1:1:1:1	1.1114

“s” denotes stress condition.

### Evaluation of derived lines for targeted and other morpho-physiological traits

The 39 BC_2_F_3_ and 21 BC_1_F_4_ families were evaluated for their performance over the recurrent parent for morpho-physiological and yield traits. The morphological traits such as plant height, spike length, and peduncle length showed little improvement over HD2733 under heat stress. However, there was a significant improvement in the derived lines for days to heading, days to maturity, tillers/plant, 1,000-kernel weight, number of spikelets/spikes, and yield/5 plants (*p* < 0.05) (Table 4). Based on the CD values at a 5% level of significance for individual traits (Table 4), it was found that all selected 39 BC_2_F_3_ lines performed better or at par with HD2733 for most of the traits. There was an improvement of 4.3% and 35.5% for the number of spikelets/spike and yield/5 plants, respectively, over the recurrent parent. Likewise, most of the selected BC_1_F_4_ lines were also superior or similar to the recurrent parent for a majority of traits. An improvement of 8.4% for 1,000-kernel weight and 18.8% for yield/5 plants over the recurrent parent was observed (Table 4). We observed a 32% reduction in the number of grains/5 spikes in HD2733 under the stress condition; however, the donor parent was not much affected by this trait under stress. The selected BC_2_F_3_ and BC_1_F_4_ lines showed ∼6% improvement for this trait over HD2733 under heat stress. Yield, the most important trait, was found to have a reduction of 16.5% in HD2733 when subjected to stress, but the improved lines showed better performance than the recipient parent. BC_2_F_3_ lines showed an increase of 35.5%, and BC_1_F_4_ lines showed 18.8% increase in yield under stress over the recurrent parent.

**TABLE 4:** Morpho-physiological trait observations of recurrent parent and derived lines under heat stress.

	HD2733 (s)	BC_2_F_3_ (%gain)	BC_1_F_4_ (%gain)	T test	*p*-value	CD (5%)
DH	87	83 (4.5%)	80 (8%)	−3.04	0.004	4.3
DM	122	116 (4.9%)	113 (7.3%)	−3.44	0.001	2.86
CT	19.84	19.5 (1.7%)	20.55	3.95	0	1.47
NDVI	0.8	0.78	0.79	6.06	0	0.036
%GC	21.85	20.93	23.72 (8.5%)	7.48	0	1.66
MSI	172.44	287.95 (66.9%)	199.91 (15.9%)	−4.43	0	14.21
SC	355.5	324.33	387.02 (8.8%)	2.89	0.005	39.95
ChL content	49.51	51.03 (3%)	48.71	−4.13	0	0.84
Spk/sp	18.4	19.2 (4.3%)	18.6 (1%)	−2.41	0.019	0.49
Seeds/5spikes	191.5	204.4 (6.7%)	203 (6%)	−0.10	0.922	27.94
Tillers/pl	13.2	13.6 (3%)	11.2	−3.8	0	1.35
TKW	42.46	40.9	46.04 (8.4%)	2.7	0.009	3.86
Yield/5 pl	47.43	64.3 (35.5%)	56.35 (18.8%)	−2.45	0.017	3.71

Figures in parenthesis depict the percentage gain over the recurrent parent during stress (s).

An evaluation of physiological traits such as canopy temperature (CT), stomatal conductance (SC), normalized difference vegetation index (NDVI), and chlorophyll content was carried out at different stages of the crop period (late-boot, early milky, and late milky stages). Significant improvement over the recurrent parent was observed at the early milk stage for these traits. Percentage improvement ranged from 1.7 for CT to 66.9 for membrane stability index (MSI) in the derived lines. For NDVI and per cent ground cover, the majority of the derived lines were similar to the recurrent parent. A few lines were superior to the recipient parent for chlorophyll content (3%) and SC (8.8%) (Table 4). Membrane stability index, which measures the lipid unsaturation, showed a highly significant improvement (36 lines showed absolutely better performance than HD2733) over the recipient parent under heat-stress conditions, indicating its strong association with the transferred QTLs or with other physiological traits.

The extent of the relative contribution of different physiological traits to abiotic stress tolerance was determined by significant correlations with yield under stress. Positive correlation (*p* > 0.01) with yield under stress was observed for tillers/plant (0.379**), days to anthesis (0.452**), and NDVI at the early milk stage (0.399**). Canopy temperature measured during the early milk stage (−0.686**) showed significant but negative correlation with yield (Supplementary Table S2). The performance of the progenies in the late sown conditions revealed a significant variance for the majority of the traits. The treatment mean sum of squares of the derived lines is provided as an online resource (Supplementary Table S3). The QTL *per se* performance of the derived lines carrying either single or both QTLs for the targeted traits is presented in Table 5. Lines pyramided with both QTLs and those carrying single QTL for DA revealed early heading, anthesis, and maturity in comparison to the lines carrying single QTL for yield under stress. However, the derived lines performed better in yield, irrespective of having either sole QTL for DA and yield or having pyramided QTLs.

**TABLE 5:** Morpho-physiological characters of parents and selected MABB-derived lines with single and two QTLs.

Traits	HD2733 (s)	WH730 (s)	BC_2_F_3_			BC_1_F_4_		
			*qANTH + qYield(s)*	*qANTH*	*qYield (s)*	*qANTH + Yield(s)*	*qANTH*	*qYield (s)*
FLE	80	70	72.12	71.28	79.73	71.44	71.16	72.5
DH	87	76	80.87	79.42	85.34	80.22	80.41	81.41
DA	91	81	84.75	83	90.17	83.88	83.91	85.5
DM	122	110	114.12	113.64	118.6	113.44	112.91	116.83
Spikelets/spike	18.4	19.6	20.05	18.9	19.1	18.85	18.67	18.53
Seeds/5 spike	192	217	230.06	198.89	213.5	196.83	192.41	214.2
Biomass/5 pl	150	140	267.5	201.42	272.6	156.66	179.16	158.33
1000kwt	42.5	45.5	33.7	43.49	42.34	46.025	45.5	46.72
HI	31.6	37.6	25.48	28.86	30.22	37.17	29.58	41.12
Yield/5 plant	47.4	52.7	59.35	54.17	58.61	58.53	51.36	57.9
CT_gf_	23.53	25.85	23.72	23.1	23.3	23.56	23.31	23.66
NDVI_gf_	0.63	0.53	0.62	0.61	0.64	0.58	0.56	0.56
MSI	172.44	318.12	266.00	220.04	264.92	217.22	182.58	254.13
Chl content	40.15	37.13	38.37	39.76	41.24	38.15	38.13	33.43

“s” denotes stress condition.

Among the 39 BC_2_F_3_ and 21 BC_1_F_4_ families, ten lines carrying single QTL for DA (81–85 days) were early in anthesis and superior in grain yield (52.03–56.94) than the recipient parent (91 days and 47.43 gms, respectively). Nine BC_2_F_3_ and three BC_1_F_4_ families carrying single QTL for yield under stress revealed a significant improvement over the recurrent parent; however, there was little improvement for days to anthesis (83–94) in these lines. The five families pyramided with both QTLs had early anthesis (83–85 days) and superior grain yield under stress (55.07–96.13 gms). Furthermore, a total of 27 (17 BC_2_F_3_ and 10 BC_1_F_4_) lines were carried forward for multi-location testing.

### Genomic contribution of donor parent regions and agronomic evaluation of improved lines

**FIGURE 2:**
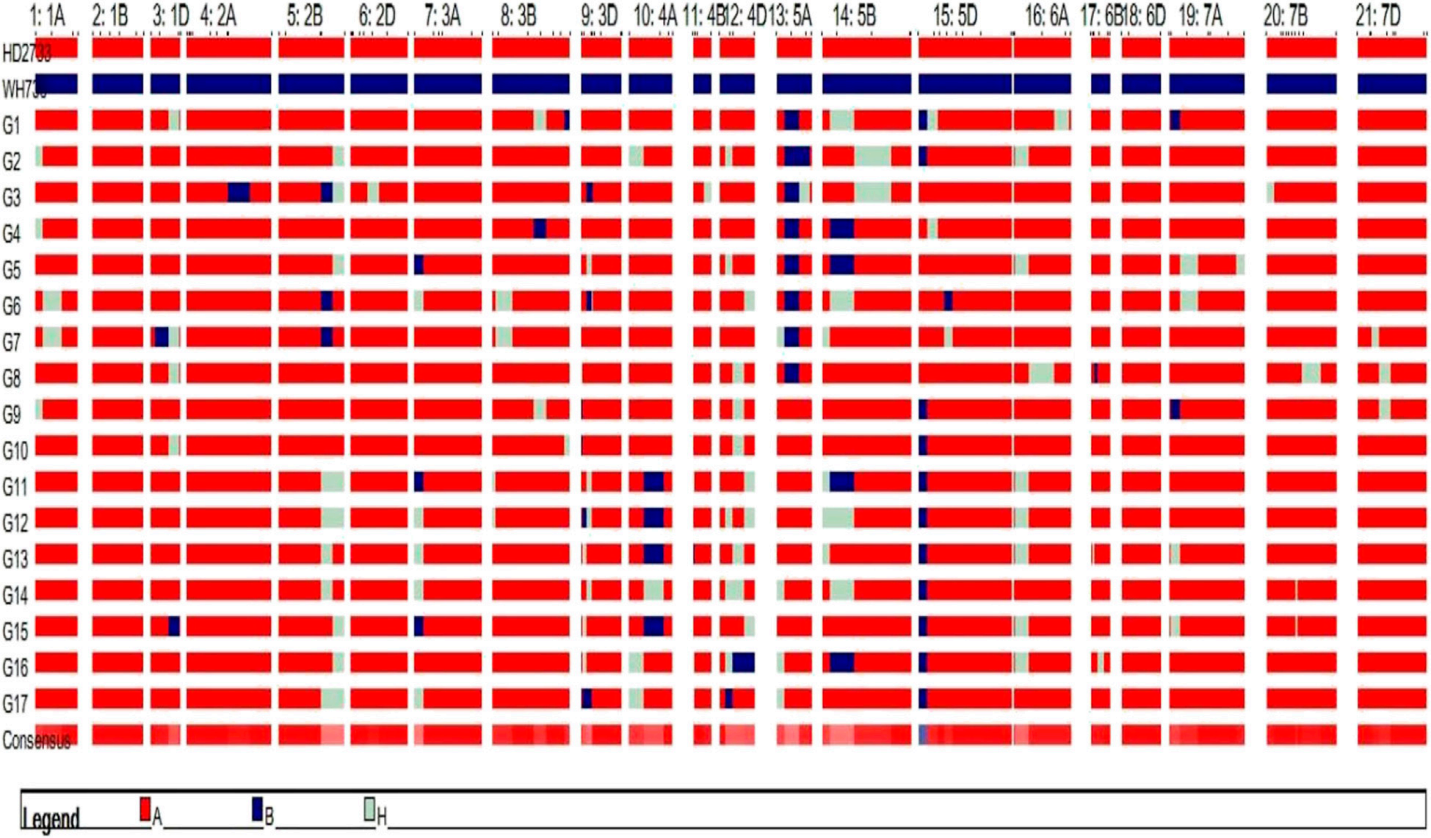
Graphical genotyping image of the selected 17 BC_2_F_3_ MABB lines. Red and blue represent the genomes of RP and DP genomic regions, respectively, and residual heterozygous regions are represented in white.

**FIGURE 3:**
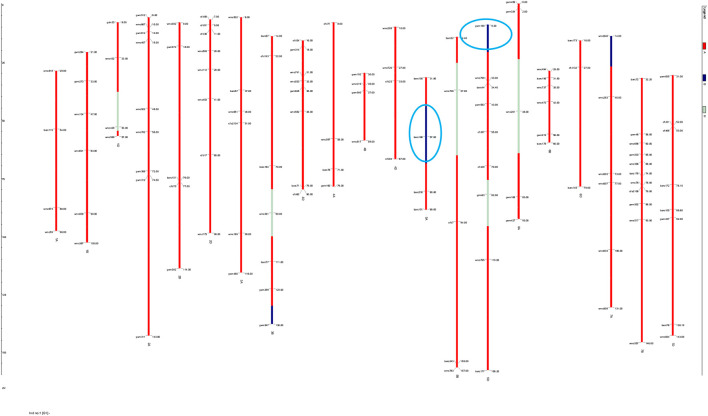
Graphical genotyping image of the best selected line with maximum genome recovery of RP and carrying both targeted QTLs.

The substituted chromosomal segment of donor parent WH730 was in the range of 0.0%–3.2% in BC_2_F_3_ and BC_1_F_4_ selected lines excluding the introgressed targeted regions on 5A and 5D chromosomes (Table 6). The graphical genotyping image for 17 selected BC_2_F_3_ lines showed the targeted QTL-linked X*barc186* and X*gwm190* markers, the maximum recovery of the recurrent parent genome at non-targeted regions on carrier chromosomes, and residual minimum donor parent regions (Figure 2). The line HD2733-210-45-812-3 with the highest percent of genome recovery (96.7%) transferred with both QTLs is represented in Figure 3.

**TABLE 6:** Performance of the targeted traits and genomic constitution of MABB-derived lines.

Genotypes	QTL	DA	Yield/5plant (gms)	DA	Yield/20 spikes (gms)	RPG %	HD2733 allele %	Heterozygous allele %	WH 730 allele %
HD2733	--	91	47.43	76	23.6	--	--	--	--
WH730	q ANTH+ q YIELD	80	52.65	67	25.74	--	--	--	--
				BC_2_F_3_	BC_2_F_4_				
HD2733-129-44-246-1	q ANTH+ q YIELD	84	96.13	75	23.29	96.3	94.3	4	1.6
HD2733-210-45-812-3	q ANTH+ q YIELD	83	78.53	73	26.35	96.7	94.3	4.8	0.8
HD2733-129-44-241-7	q ANTH	83	56.23	73	25.73	93.9	91.1	5.6	3.2
HD2733-210-45-777-8	q ANTH	83	52.18	69	33.38	97.5	96.7	1.6	1.6
HD2733-210-45-777-9	q ANTH	82	56.94	72	25.94	95.9	93.5	4.8	1.6
HD2733-154-152-314-14	q ANTH	81	54.81	69	23.55	94.7	91.9	5.6	2.4
HD2733-182-156-398-15	q ANTH	81	55.42	—	—	95.5	92.7	5.6	1.6
HD2733-210-42-756-16	q ANTH	84	56.08	68	29.49	96.7	94.3	4.8	0.8
HD2733-129-44-220-20	q YIELD	89	75.73	72	27.06	96.7	95.1	3.2	1.6
HD2733-129-44-225-21	q YIELD	92	79.71	73	30.5	97.9	96.7	2.4	0.8
HD2733-210-31-908-26	q YIELD	92	80.74	71	27.63	94.3	91.1	6.4	2.4
HD2733-210-31-910-27	q YIELD	90	79.06	70	30.19	94.3	90.3	8	1.6
HD2733-210-31-915-28	q YIELD	90	85.19	70	23.42	95.1	91.9	6.4	1.6
HD2733-210-31-916-29	q YIELD	90	80.48	71	24.85	96.3	92.7	7.2	0
HD2733-210-31-938-32	q YIELD	94	78.7	73	27.73	95.1	91.1	5.6	3.2
HD2733-210-31-948-35	q YIELD	89	69.53	73	29.42	94.3	91.1	6.4	2.4
HD2733-210-31-959-37	q YIELD	90	75.01	72	36.56	95.1	91.1	7.2	1.6
			BC_1_F_4_	BC_1_F_5_					
HD2733-154-46-3	q ANTH+ q YIELD	84	71.68	70	33.61	92	86.4	11.2	2.4
HD2733-182-81-4	q ANTH+ q YIELD	83	55.07	69	24.33	92	88	8	3.2
HD2733-485-153-6	q ANTH+ q YIELD	85	71.45	74	22.03	92.4	86.4	12	1.6
HD2733-210-209-11	q ANTH	85	52.03	69	29.42	92.4	85.6	13.6	0.8
HD2733-210-209-12	q ANTH	82	53.32	72	22.85	92.4	88	8.8	3.2
HD2733-210-209-13	q ANTH	82	53.22	73	20.46	92.8	87.2	11.2	1.6
HD2733-210-209-16	q ANTH	85	52.75	—	—	92.4	86.4	12	1.6

The selected 17 BC_2_F_3_ and 10 BC_1_F_4_ families were advanced to successive generation and subjected to agronomic evaluation under late sowing condition. Furthermore, eight lines were selected for multi-location yield trials in the next season (Table 6) on the basis of superior yield in comparison to the recurrent parent under stress conditions. The selected eight lines were evaluated in large plots at three locations, namely, New Delhi, Bihar, and Pune, which resulted in the final selection of three superior lines for further entry into a varietal testing system for release (Table 7).

**TABLE 7:** Multi-location evaluation of the selected homozygous NILs.

Lines	QTL	Pusa Bihar		Delhi RI		Delhi LS		Pune	
		Yield (q/ha)	DA	Yield (q/ha)	DA	Yield (q/ha)	DA	Yield (q/ha)	DA
HD2733 NIL15	*qANTH + qYield(s)*	58.4	75	50.83	81	35.97	66	48.47	64
HD2733 NIL23	*qANTH*	68.5	76	53.61	82	38.06	65	26.07	68
HD2733 NIL6	*qYield (s)*	34.34	85	46.53	87	30.42	67	35.1	76
HD2733	—	57.27	85	46.53	91	25.69	72	22.98	73
CD 5%	—	0.87	—	1.72	—	3.21	—	2.28	—

Delhi LS: Delhi late sown; Delhi RI: restricted irrigation stress with one irrigation only.

## Discussion

Heat stress is a limiting factor in the global agricultural production by preventing the crop from its potential genetic yield. To develop tolerance to heat stress, improvement of wheat varieties with stress-tolerant genes/QTLs is the most effective strategy. MABB is considered as one of the reliable methods to improve a crop variety by incorporating the desired gene(s)/QTLs that govern the trait expression in which the variety is essentially deficient. Numerous reports are available on molecular markers linked with the expression of QTLs for heat-stress tolerance (Devate et al., 2022; Khan et al., 2022; Pinto et al., 2010; Gupta et al., 2012; Gupta et al., 2017) but their use in wheat-breeding programs is still rare. The present study is an attempt of the transfer of QTLs associated with heat stress in to the background of high-yielding wheat varieties using MABB.

Marker-assisted foreground selection had been used successfully in earlier studies (Alam et al., 2012; Singh et al., 2012; Babu et al., 2017; Rai et al., 2018; Todker et al., 2020; Pandit et al., 2021) for abiotic stress such as identification of salt-tolerant genotypes in rice (Neerja et al., 2007) and for biotic stress as downy mildew resistance in bajra (Hash et al., 2006). In the present study, foreground selection helped to select only those desirable genotypes that were carrying the QTLs (either in homozygous or heterozygous) for targeted morpho-physiological traits imparting tolerance to the heat stress.

It has been found that the applications of background selection with genome-wide polymorphic markers hasten the RPG recovery in MABB (Servin and Hospital, 2002; Chen et al., 2008; Basavaraj et al., 2010). The simulation studies on marker-assisted breeding (Hospital et al., 1992; Hospital, 2003; Servin et al., 2004) recommended that a minimum of four markers per chromosome (2 markers on each arm) at an average distance of 20 cM between markers is sufficient for the accelerated recovery of the recipient parent genome with a sufficient population size. Hence, marker alleles corresponding to HD2733 were selected for background screening to determine the actual recovery of RPG in the early segregating generations, making it possible to reduce the number of genotypes to be carried to the next generation. Selected plants had an enhanced RPG recovery ranging from 67.3% to 75.4% in BC_1_F_1_ and 83.33% to 94.44% in BC_2_F_1_. This additional recovery is due to the fixation of recipient allele from a heterozygous condition which may be theoretically gained after 3–4 backcrossing in case of single-gene/QTL transfer which also could have taken an additional number of backcrossing in case of more than two QTLs/gene pyramiding.

Multiple QTL mapping studies performed over the years have identified several QTLs associated with physiological, morphological, and agronomic traits in wheat (Griffths et al., 2009; Griffths et al., 2012; Gupta et al., 2017; Puttamadanayaka et al., 2020). Meta-analysis of such QTLs identified genomic regions that contribute to improved adaptation under stress (Acuna-Galindo et al., 2015). In earlier studies, there were 43 meta-QTL (MQTL) regions that co-localized with traits governing both drought and heat stress. MQTL38 on 5A chromosome harbors individual QTL for days to heading, biomass, CT, maturity, stay-green, yield, kernel number, and harvest index (Acuna-Galindo et al., 2015). The present study reports the transfer of the X*barc186* marker that co-localized with the MQTL38 region, known for drought and heat stress-adaptive traits. The improved lines were superior in performance probably due to introgression of this meta-QTL region governing beneficial genes for heat-tolerant traits. Another QTL-linked SSR marker X*gwm190* for grain yield under stress lies on chromosome 5D according to the high-density consensus linkage map (Somers et al., 2004). However, Mohammadi et al. (2008) reported the presence of the X*gwm190* marker on 1D chromosome in its linkage map. Blast analysis of the sequence of marker X*gwm190* with *Triticum aestivum* genome sequence (www.ensemblplants.org/triticum aestivum/release 47) revealed its location at the 5D: 8746873-8747085 region. The linkage mapping carried out earlier in our lab for heat tolerance also determined *Xgwm190* position at the upper arm of 5D (Sun et al., 2021). The chromosomal location of X*gwm190* on linkage group 5D was, therefore, considered and used for transfer of the linked trait in this study. The location of X*gwm190* was very close to a trait-linked DART marker on 5D, identified for drought-tolerance in wheat in previous studies (McIntyre et al., 2010).

Tolerance to high-temperature stress is achieved by an interaction between several physiological, biochemical, and molecular components in wheat (Shashikumara et al., 2022b). High correlation between physiological traits and grain yield was observed under stress condition (Sun et al., 2021). Previous researchers reported that several morpho-physiological traits significantly contributed to yield improvement under stress and could effectively be used in breeding programs (Richards et al., 2000; Ramya et al., 2015; Cossani and Reynolds 2012; Puttamadanayaka et al., 2020; Goel et al., 2019; Yadav et al., 2006). These include traits for canopy establishment and architecture, photosynthesis, and partitioning of total assimilates to grain. In the present study, traits such as canopy temperature, membrane stability index, and stomatal conductance were improved in the derived lines introgressed with heat-tolerant QTLs. This suggests the effectiveness in use of such traits as the selection criteria in wheat breeding (Ramya et al., 2021). Spikelet fertilization and grain setting are the most critical stages sensitive to the high-temperature stress at the mid-anthesis stage (Ferris et al., 1998; Ullah et al., 2022). In the present study, under late sowing condition, HD2733 and WH730 took 91 and 80 days for anthesis, respectively. This difference of nearly 10 days subjected HD2733 to heat stress. The grain yield reduced drastically in HD2733 from 56.83 g under normal condition to 47.43 g under late sowing condition, which accounts for nearly 18.30% reduction as compared to WH730 which showed 8.01% higher yield under stress. A significant reduction of 32.86% for seeds per five spikes in HD2733 in comparison to WH730 which showed a reduction of 3.55% under stress suggesting that a decrease in grain number per spike could be one of the reasons to have a significant reduction in the grain yield of HD2733 under high temperature and improving wheat for this trait would be worthwhile for the development of tolerant varieties.

Many of the previous studies indicated a severe effect of high temperature at phenological stages, in particular, heading on seeds/spike and thereby on grain yield (Wardlaw et al., 1989; Wardlaw and Moncur, 1995; Sarker et al., 2021). Hence, early heading is a desirable trait to combat heat stress in wheat. Several cultivars had been released for adaptation to production systems to avoid reproductive stage-heat stress. The early heading lines developed in the present study can adapt to heat stress with higher yields. Early heading lines complete the initial seed setting and grain filling before the incidence of heat stress. In the eastern Gangetic plains of South-Asia, early heading had been suggested as a good approach for wheat-breeding (Joshi et al., 2007; Mondal et al., 2016). In an earlier study by Tewolde et al. (2006), it was found that early-heading wheat cultivars yielded better results than later-heading cultivars in heat stress environments, even in durum lines (Akter and Islam, 2017).

There are numerous examples of QTLs mapped for heat-tolerant traits, but the mobilization of mapped QTLs into practical breeding is extremely worthwhile. Validation of identified QTLs with high PVE (phenotypic variation explained) in different genetic backgrounds is essential for their utilization. The QTLs used in this study were mapped with high phenotypic variance for the trait of interest. The selected lines containing QTLs for days to anthesis exhibited earliness in anthesis causing early maturity to avoid the effect of heat stress without affecting grain yield. It is pertinent that early-heading wheat varieties have an adaptive mechanism to heat stress with shorter life cycles in the area where there are frequent occurrences of terminal heat stress (Mondal et al., 2016). The heat-tolerant early heading varieties had high grain-filling duration and lower senescence of leaf compared to late-heading varieties. Negative association between days to heading and grain yield has been observed in five years of South-Asian trials of early maturing varieties which support the fact that earliness enabled tolerance to high-temperature stress. The study, therefore, reports the first successful incorporation of QTLs for early heading and yield traits into the background of a high-yielding elite cultivar, HD2733. Eight homozygous lines comprising tolerant QTLs were subjected to multi-location testing, and three lines were finally identified for their subsequent entry into varietal release system.

## Conclusion

The present study improved the performance of one of the most popular wheat cultivars, HD2733, by introgression of QTLs associated with early anthesis and high-kernel weight under high-temperature stress. The study has led to the development of MABB-derived lines with targeted QTLs that caused earliness, and plants escaped the terminal stage heat stress without compromising the grain yield. These derived genotypes were further advanced for multi-location testing, and three lines were finally selected for subsequent release as improved varieties. The lines may also serve as the best genetic materials for functional genomics and expression studies to understand the molecular pathways and mechanisms underlying the stress tolerance governed by the respective QTLs without the effect of background noise. Furthermore, improved HD2733 can be used as genetic resource for the wheat-breeding program for heat-stress tolerance.

## Data Availability

The raw data supporting the conclusion of this article will be made available by the authors, without undue reservation.
