# Supplementary material for: Marker-assisted backcross breeding for heat tolerance in bread wheat (Triticum aestivum L.)
Source: Front Genet. 2022 Dec 8;13:1056783. doi: 10.3389/fgene.2022.1056783 (PMC9785257; doi:10.3389/fgene.2022.1056783)
Supplement: Supplementary file 1 [file Table1.DOC]

**List of SSR primers used for Background selection**

**Supplementary table 1: List of SSR markers used in background selection**

| S. No. | Primer name | Forward | Reverse | Annealing temp. ͦ C |
| --- | --- | --- | --- | --- |
| 1 | wmc 818 | TGAAGGGTGCGTGTGGTC | GCGTCGATTTTAATTTGATGATGG | 61 |
| 2 | barc119 | CACCCGATGATGAAAAT | GATGGCACAAGAAATGAT | 55 |
| 3 | wmc673 | AGGAAACAAGAGTGTGTGTGGG | AGGAATAAGGACTCGCAAAACG | 61 |
| 4 | wmc59 | TCATTCGTTGCAGATACACCAC | TCAATGCCCTTGTTTCTGACCT | 61 |
| 5 | gwm273 | ATTGGACGGACAGATGCTTT | AGCAGTGAGGAAGGGGATC | 55 |
| 6 | wmc134 | CCAAGCTGTCTGACTGCCATAG | AGTATAGACCTCTGGCTCACGG | 61 |
| 7 | wmc631 | TTGCTCGCCCACCTTCTACC | GGAAACCATGCGCTTCACAC | 61 |
| 8 | wmc830 | ACCTTTTCCTGCATCGGCT | CTCCGCTCGTGTCCAACTATC | 61 |
| 9 | wmc367 | CTGACGTTGATGGGCCACTATT | GTGGTGGAAGAGGAAGGAGAGG | 61 |
| 10 | gdm33 | GGCTCAATTCAACCGTTCTT | TACGTTCTGGTGGCTGCTC | 60 |
| 11 | wmc432 | ATGACACCAGATCTAGCAC | AATATTGGCATGATTACACA | 51 |
| 12 | wmc429 | ATGACACCAGATCTAGCAC | AATATTggcATgATTAcAcA | 51 |
| 13 | wmc590 | CGCACGAAGCTATCTGATACCA | GGAAAACCTAACCCTAGCCACC | 61 |
| 14 | gwm512 | AGCCACCATCAGCAAAAATT | GAACATGAGCAGTTTGGCAC | 60 |
| 15 | wmc667 | GAGGAGAGGAAAAGGCAGGCTA | AACTCTTGCGTGTCTCAAACCG | 61 |
| 16 | gwm614 | GATCACATGCATGCGTCATG | TTTTACCGTTCCGGCCTT | 60 |
| 17 | wmc407 | GGTAATTCTAGGCTGACATATGCTC | CATATTTCCAAATCCCCAACTC | 61 |
| 18 | wmc522 | AAAAATCTCACGAGTCGGGC | CCCGAGCAGGAGCTACAAAT | 61 |
| 19 | wmc702 | GAATCACATCGAATGGATCTCA | GAGGCCTTTTTCGATATTCTGC | 61 |
| 20 | gwm312 | ATCGCATGATGCACGTAGAG | ACATGCATGCCTACCTAATGG | 60 |
| 21 | gwm311 | TCACGTGGAAGACGCTCC | CTACGTGCACCACCATTTTG | 60 |
| 22 | wmc382 | CATGAATGGAGGCACTGAAACA | CCTTCCGGTCgACGCAAC | 61 |
| 23 | gwm374 | ATAGTGTGTTGCATGCTGTGTG | TCTAATTAGCGTTGGCTGCC | 60 |

| S. No. | Primer name | Farward | Reverse | Annealing Temp. ͦ C |
| --- | --- | --- | --- | --- |
| 24 | barc101 | GCTCCTCTCACGATCACGCAAAG | GCGAGTCGATCACACTATGAGCCAATG | 52 |
| 25 | cfd70 | GTCGGCATAGTCGCACATAC | ACTATGCCAAGGGGAGTGTG | 60 |
| 26 | gwm382 | GTCAGATAACGCCGTCCAAT | CTACGTGCACCACCATTTTG | 60 |
| 27 | cfd56 | TTGCATAATTACTTGCCCTCC | CTGGTCCAACTTCCATCCAT | 60 |
| 28 | cfd51 | GGAGGCTTCTCTATGGGAGG | TGCATCTTATCCTGTGCAGC | 60 |
| 39 | cfd36 | GCAAAGTGTAGCCGAGGAAG | TTAGAGTTTTGCAGCGCCTT | 60 |
| 30 | wmc503 | GCAATAGTTCCCGCAAGAAAAG | ATCAACTACCTCCAGATCCCGT | 61 |
| 31 | wmc112 | TGAGTTGTGGGGTCTTGTTTGG | TGAAGGAGGGCACATATCGTTG | 61 |
| 32 | wmc453 | ACTTGTGTCCATAACCGACCTT | ATCTTTTGAGGTTACAACCCGA | 61 |
| 33 | cfd17 | AGCACAGAAGGGGTTAGGGT | AGCTGCGGTGTGAGCTAAAT | 60 |
| 34 | wmc175 | gcTcAgTcAAAccgcTAcTTcT | cAcTAcTccAATcTATcgccgT | 61 |
| 35 | wmc532 | gATAcATcAAgATcgTgccAAA | gggAGAAATCATTAACGAAGGG | 61 |
| 36 | barc57 | GCGACCACCTCAGCCAACTTATTATGT | GCGGGGAGGCACATTCATAGGAGT | 55 |
| 37 | wmc651 | CGACGACGTCCGGGTG | CATTTCCTCTCCCATATCTCTCATC | 61 |
| 38 | cfa2134 | TTTACGGGGACAGTATTCGG | AAGACACTCGATGCGGAGAG | 60 |
| 39 | wmc169 | TACCCGAATCTGGAAAATCAAT | TGGAAGCTTGCTAACTTTGGAG | 61 |
| 40 | gwm480 | GCTCACCGGGCATTGGGATCA | GCGATGACGAGATAAAGGTGGAGAAC | 55 |
| 41 | cfd143 | TTCTCCATGGGCAGCTACTT | ACTACTTGCGGACGGCTG | 60 |
| 42 | barc164 | TGCAAACTAATCACCAGCGTAA | CGCTTTCTAAAACTGTTCGGGATTTCTAA | 50 |
| 43 | wmc291 | TACCACGGGAAAGGAAACATCT | CACGTTGAAACACGGTGAcTAT | 61 |
| 44 | BARC77 | GCGTATTCTCCCTCGTTTCCAAGTCTG | GTGGGAATTTCTTGGGAGTCTGTA | 55 |
| 45 | cfd34 | GGAAGAACCGCAACAGACAT | GCATCTTCTCCTCCCTCCTC | 60 |

| S. No. | | Primer name | | Farward | | Reverse | | Annealing temp. ͦ C | |  |
| --- | --- | --- | --- | --- | --- | --- | --- | --- | --- | --- |
|  | 46 | | wmc741 | | CAACAACGCTAGAGGCCAAC | | GGGCTCCATGCTCTTCC | | 61 | |
|  | 47 | | wmc533 | | AATTGGATCGGCAGTTGGAG | | AGCAAGCAGAGCATTGCGTT | | 61 | |
|  | 48 | | gwm645 | | TGACCGGAAAAGGGCAGA | | GCCCCTGCAGGAGTTTAAGT | | 55 | |
|  | 49 | | wmc552 | | ACTAAGGAGTGTGAGGGCTGTG | | CTCTCGCGCTATAAAAGAAGGA | | 61 | |
|  | 50 | | barc71 | | GCGCTTGTTCCTCACCTGCTCATA | | GCGTATATTCTCTCGTCTTCTTGTTGGTT | | 55 | |
|  | 51 | | cfd62 | | CAAGAGCTGACCAATGTGGA | | ACGGCGGTGAGATGAG | | 60 | |
|  | 52 | | wmc597 | | AACACACCTTGCTTCTCTGGGA | | GACTAGGGTTTCGGTTGTTGGC | | 61 | |
|  | 53 | | BARC78 | | CTCCCCGGTCAAGTTTAATCTCT | | GCGACATGGGAATTTCAGAAGTGCCTAA | | 55 | |
|  | 54 | | gwm160 | | TTCAATTCAGTCTTGGCTTGG | | CTGCAGGAAAAAAAGTACACCC | | 60 | |
|  | 55 | | gwm192 | | GGTTTTCTTTCAGATTGCGC | | CGTTGTCTAATCTTGCCTTGC | | 60 | |
|  | 56 | | wmc419 | | GTTTCGGATAAAACCGGAGTGC | | ACTACTTGTGGGTTATCACCAGCC | | 61 | |
|  | 57 | | gwm540 | | TCTCGCTGTGAAATCCTATTTC | | AGGCATGGATAGAGGGGC | | 55 | |
|  | 58 | | wmc617 | | CCACTAGGAAGAAGGGGAAACT | | ATCTGGATTACTGGCCAACTGT | | 61 | |
|  | 59 | | wmc285 | | TGTGGTTGTATTTGCGGTATGG | | TTGTGGTGCTGAGTTAGCTTGT | | 61 | |
|  | 60 | | wmc720 | | CACCATGGTTGGCAAGAGA | | CTGGTGATACTGCCGTGACA | | 61 | |
|  | 61 | | cfd23 | | TAGCAGTAGCAGCAGCAGGA | | GCAAGGAAGAGTGTTCAGCC | | 60 | |
|  | 62 | | cfd84 | | GTTGCCTCGGTGTCGTTTAT | | TCCTCGAGGTCCAAAACATC | | 60 | |
|  | 63 | | barc100 | | CCGTTAAGCCGCCTACCACAGAGTTGC | | GGCTTAAAACGGATCCTCCAGGTCAT | | 52 | |
|  | 64 | | barc186 | | GGAGTGTCGAGATGATGTGGAAAC | | CGCAGACGTCAGCAGCTCGAGAGG | | 58 | |
|  | 65 | | barc230 | | CC CTC CTC CTT CTC CCT CCT CCT A | | GGC TCA TGC GGG CGT GTT TGG | | 58 | |
|  | 66 | | barc151 | | TGAGGAAAATGTCTCTATAGCATCC | | CGCATAAACACCTTCGCTCTTCCACTC | | 55 | |
|  | 67 | | barc32 | | GCGTGAATCCGGAAACCCAATCTGTG | | TGGAGAACCTTCGCATTGTGTCATTA | | 52 | |

| S. No. | Primer name | Farward | Reverse | Annealing temp. ͦ C |
| --- | --- | --- | --- | --- |
| 68 | wmc728 | GCAGGCTCTGCATCTTCTTG | CGCAGAGCTGAGCTGAAATC | 61 |
| 69 | cfd7 | AGCTACCAGCCTAGCAGCAG | TCAGACACGTCTCCTGACAAA | 60 |
| 70 | barc243 | CGCAAAATCGAAATTAAAAATGGAAA | GATCCTCCTTTCAGCTGGCCTATTA | 50 |
| 71 | wmc783 | AGGTTGGAGATGCAGGTGGG | TCTTCCTTCTCCTGCCGCTA | 61 |
| 72 | gwm190 | GTGCTTGCTGAGCTATGAGTC | GTGCCACGTGGTACCTTTG | 60 |
| 73 | wmc799 | CGTACGTACGCCTGTACCCTTG | AATCTTGGGCGTCTAATCTTTTGC | 61 |
| 74 | barc44 | CCCTACAAAATACGAACATGAAGTCAG | GGGTCCTACTCAGATAGTGACAGTCAAC | 50 |
| 75 | gwm583 | TTCACACCCAACCAATAGCA | TCTAGGCAGACACATGCCTG | 60 |
| 76 | cfd57 | ATCGCCGTTAACATAGGCAG | TCACTGCTGTATTTGCTCCG | 60 |
| 77 | cfd29 | GGTTGTCAGGCAGGATATTTG | TATTGATAGATCAGGGCGCA | 60 |
| 78 | gdm63 | GCCCCCTATTCCATAGGAAT | CCTTTTGATGGTGCATAGGA | 60 |
| 79 | wmc765 | GGGATCAGACTGGGACTGGAG | GGGTTGGCTTGGCAGAGAA | 61 |
| 80 | gwm459 | ATGGAGTGGTCACACTTTGAA | AGCTTCTCTGACCAACTTCTCG | 55 |
| 81 | gwm334 | AATTTCAAAAAGGAGAGAGA | AACATGTGTTTTTAGCTATC | 50 |
| 82 | wmc201 | cATgcTcTTTcAcTTgggTTcg | gcgcTTgcAggAATTcAAcAcT | 61 |
| 83 | gwm169 | ACCACTGCAGAGAACACATACG | GTGCTCTGCTCTAAGTGTGGG | 60 |
| 84 | gwm427 | AAACTTAGAACTGTAATTTCAGA | AGTGTGTTCATTTGACAGTT | 50 |
| 85 | wmc494 | ggATcgAgTcTcAAgTcTAcAA | AgAAggAAcAAgcAAcATcATA | 51 |
| 86 | barc198 | CGCTGAAAAGAAGTGCCGCATTATGA | CGCTGCCTTTTCTGGATTGCTTGTCA | 50 |
| 87 | wmc737 | CGACTAGGACTAGACGACTCTAACGG | GTCGATCACCAGAGGCATTG | 61 |
| 88 | wmc473 | TcTgTTgcgcgAAAcAgAATAg | cccATTggAcAAcAcTTTcAcc | 61 |

| S. No. | Primer name | Farward | Reverse | Annealing temp. ͦ C |
| --- | --- | --- | --- | --- |
| 89 | gwm219 | GATGAGCGACACCTAGCCTC | GGGGTCCGAGTCCACAAC | 60 |
| 90 | barc178 | GCGTATTAGCAAAACAGAAGTGAG | GCGACTAGTACGAACACCACAAAA | 52 |
| 91 | barc173 | GGGGATCCTTCAACAATAACA | GCGAGATGGCATTTTTAAATAAAGAGAC | 50 |
| 92 | cfd132 | CAAATGCTAATCCCCGCC | TGTAAACAAGGTCGCAGGTG | 60 |
| 93 | barc145 | GCAGCCTCGAATCACA | GGGGTGTTGAAGATGA | 52 |
| 94 | wmc646 | GGAGTAAATGGAGACGGGGAC | GCCAGTGTGATGCATGTGAC | 61 |
| 95 | wmc283 | cgTTggcTgggTTATATcATcT | gAcccgcgTgTAAgTgATAggA | 61 |
| 96 | wmc603 | ACAAACGGTGACAATGCAAGGA | CGCCTCTCTCGTAAGCCTCAAC | 61 |
| 97 | wmc607 | ATATATGCCCATGAAGCTCAAG | GATCGAGCTAAAGCTGATACCA | 61 |
| 98 | wmc633 | ACACCAGCGGGGATATTTGTTAC | GTGCACAAGACATGAGGTGGATT | 61 |
| 99 | wmc809 | CAGGTCGTAGTTGGTACCCTGAA | TGAACACGGCTGGATGTGA | 61 |
| 100 | barc72 | CGTCCTCCCCCTCTCAATCTACTCTC | CGTCCCTCCATCGTCTCATCA | 60 |
| 101 | wmc696 | ACCCGAGAGAGATTAGGGCTTG | CACTCGCAGCCTCTCTTCTACC | 61 |
| 102 | gwm333 | GCCCGGTCATGTAAAACG | TTTCAGTTTGCGTTAAGCTTTG | 55 |
| 103 | wmc396 | TGCACTGTTTTACCTTCACGGA | CAAAGCAAGAACCAGAGCCAC | 61 |
| 104 | barc176 | GCGAAAGCCATCAAACACTATCCAACT | GGTAACTAAGCACGTCACAAGCATAAA | 50 |
| 105 | wmc76 | CTTCAGAGCCTCTTTCTCTACA | CTGCTTCACTTGCTGATCTTTG | 51 |
| 106 | cfa2106 | GCTGCTAAGTGCTCATGGTG | TGAAACAGGGGAATCAGAGG | 60 |
| 107 | gwm302 | GCAAGAAGCAACAGCAGTAAC | CAGATGCTCTTCTCTGCTGG | 60 |
| 108 | wmc517 | ATCCTGACGTTACACGCACC | ACCTGGAACACCACGACAAA | 61 |
| 109 | wmc557 | GGTGCTTGTTCATACGGGCT | AGGTCCTCGATCCGCTcAT | 61 |
| 110 | gwm635 | TTCCTCACTGTAAGGGCGTT | CAGCCTTAGCCTTGGCG | 60 |
| 111 | cfd31 | GCACCAACCTTGATAGGGAA | GTGCCTGATGATTTTACCCG | 60 |
| 112 | cfd66 | AGGTCTTGGTGGTTTTGGTG | TTTTCACATGCCCACAGTTG | 60 |
| 113 | barc172 | GCGAAATGTGATGGGGTTTATCTA | GCGATTTGATTTAACTTTAGCAGTGAG | 50 |
| 114 | barc105 | CAG GAA GAA AAG GAA AGC ATG CGA CAA | GCG GTG TGG CAA TAA TTA CTT TTT | 50 |
| 115 | gwm437 | GATCAAGACTTTTGTATCTCTC | GATGTCCAACAGTTAGCTTA | 50 |
| 116 | barc76 | ATTCGTTGCTGCCACTTGCTG | GCGCGACACGGAGTAAGGACACC | 58 |
| 117 | wmc634 | AGCGAGGAGGATGCATCTTATT | GACATACACATGATGGACACGG | 61 |
| 118 | gwm264 | - | - | 60 |
| 119 | gwm388 | 5' CTACAATTCGAAGGAGAGGGG 3' | 5' CACCGCGTCAACTACTTAAGC 3' | 60 |
| 120 | barc87 | 5' GCTCACCGGGCATTGGGATCA 3' | 5' GCGATGACGAGATAAAGGTGGAGAAC 3' | 55 |
| 121 | gwm547 | - | - | 60 |
| 122 | gwm299 | - | - | 60 |
| 123 | cfd71 | 5' CAATAAGTAGGCCGGGACAA 3' | 5' TGTGCCAGTTGAGTTTGCTC 3' | 60 |
| 124 | barc177 | 5' GCGATCCTGTTGTTGAGCGTTTGCATA A 3' | 5' TCCCGTTTTCCCGTGTGTTAGTCTA 3' | 50 |

| **Supplementary Table 2:** Correlation coefficients of morpho-physiological traits with DA and yield under stress. | | | | | |
| --- | --- | --- | --- | --- | --- |
| S. No. | Traits | yield(s) | S. No. | Traits | days to anthesis |
| 1 | DH | 0.364* | 1 | FLE | 0.774** |
| 2 | DA | 0.452** | 2 | DH | 0.973** |
| 3 | Tillers/Plant | 0.379** | 3 | DM | 0.818** |
| 4 | CT(early milk) | -0.686** | 4 | Peduncle Length | -0.335** |
| 5 | NDVI (early milk) | 0.399** | 5 | Tillers/plant | 0.363** |
| 6 | Biomass/ 5pl | 0.631** | 6 | NDVI (late boot) | 0.328* |
|  |  |  | 7 | NDVI(late milk) | 0.492** |
|  |  |  | 8 | MSI | -0.442** |
|  |  |  | 9 | Chlorophyll (late milk) | 0.569** |
|  |  |  | 10 | Biomass/5 pl | 0.289** |
|  |  |  | 11 | Yield | 0.452** |

| **Supplementary Table 3:** Mean sum of squares of augmented design ANOVA for various traits in MABB derived lines. | | | |
| --- | --- | --- | --- |
| **S. No.** | **Characters** | **BC_2_F_3_ treatment MSS** | **BC_1_F_4_ treatment MSS** |
| 1 | FLE | 9.2921** | 14.1717* |
| 2 | DH | 9.1692 | 10.6010* |
| 3 | DA |  |  |
| 4 | DM | 10.2929* | 20.2626* |
| 5 | PH | 91.6894** | 22.0015* |
| 6 | SL | 0.8235 | 0.7999 |
| 7 | PL | 15.9214* | 14.1304* |
| 8 | Spk/sp | 1.1600** | 0.3052* |
| 9 | Tillers/sp | 6.8755** | 2.8224* |
| 10 | Seeds/5sp | 837.9128** | 626.3636** |
| 11 | Biomass/5pl | 7577.0643** | 1832.5757** |
| 12 | 1000kwt | 60.4833** | 592.8315* |
| 13 | HI | 50.5164** | 85.9363** |
| 14 | Yield | 402.1982** | 122.5593** |
| 15 | CT (Late boot) | 0.1042* | 0.08151 |
| 16 | CT (early milk) | 1.5270* | 0.1972 |
| 17 | CT (Late milk) | 0.3158* | 0.5162 |
| 18 | NDVI (Late boot) | 0.00023 | 0.00014 |
| 19 | NDVI (early milk) | 0.00015 | 0.00017 |
| 20 | NDVI (Late milk) | 0.00151 | 0.003 |
| 21 | %GC | 3.7559* | 101.7331** |
| 22 | MSI | 6299.7879** | 4913.6283** |
| 23 | SC (late boot) | 22332.668* | 17262.5 |
| 24 | SC (early milk) | 5283.9264** | 7782.1926* |
| 25 | Chl (Late boot) | 6.6441** | 6.127 |
| 26 | Chl (Early milk) | 4.8708** | 3.7160* |
| 27 | Chl (Late milk) | 4.3768* | 17.9917* |

April month

January month

April month

January month

April month

January month

April month

January month

ix
